# A de novo 10q11.23q22.1 deletion detected by whole genome mate-pair sequencing: a case report

**DOI:** 10.1186/s12887-021-02723-y

**Published:** 2021-05-31

**Authors:** Dalin Fu, Weisheng Lin, Fen Lu, Senjie Du, Min Zhu, Xiaoke Zhao, Jian Tang, Chuan Chen, Xiaoli Chui, Shanmei Tang, Kai Wang, Chuanchun Yang, Bei Han

**Affiliations:** 1 Department of rehabilitation, Nanjing Children’s Hospital Affiliated to Nanjing Medical University, Nanjing, China; 2 CheerLand Precision Biomed Co., Ltd, Shenzhen, China; 3 Department of Pediatric Endocrinology, Nanjing Children’s Hospital Affiliated to Nanjing Medical University, Nanjing, 210008 China

**Keywords:** Whole-genome mate-pair sequencing, 10q11-q22 deletion syndrome, Developmental delay, Congenital cleft palate

## Abstract

**Background:**

Interstitial deletions of chromosome band 10q11-q22 was a genomic disorder distinguished by developmental delay, congenital cleft palate and muscular hypotonia. The phenotypes involved were heterogeneous, hinge on the variable breakpoints and size.

**Case presentation:**

Here, we presented a patient with soft palate cleft, growth and development delay. The patient was a 2 years and 5 months girl who was not able to walk unless using a children’s crutches to support herself. Whole-exome sequencing (WES) and whole-genome mate-pair sequencing (WGMS) were both performed by next generation sequencing (NGS). A 20.76 Mb deletion at 10q11.23q22.1 (seq[GRCh37/hg19]del(10)(50,319,387-71,083,899) × 1) was revealed by the WGMS, which was verified as de novo by quantitative polymerase chain reaction (QPCR).

**Conclusion:**

Children with 10q11-q22 deletions greater than 20 MB have never been reported before, and we are the first to report and provide a detailed clinical phenotype, which brings further knowledge of 10q11-q22 deletions.

## Background

Chromosome bands 10q11-q22 interstitial deletions were very rare and ranged in size from 0.3 to 21 Megabytes (Mb). Patients with 10q11-q22 deletions suffered from language delay, low weight, short stature, hypotonia, bilateral undescended testes, developmental delay, hypoplastic labia minora, strabismus, systolic murmur, and craniofacial dysmorphisms such as anteverted nares, broad-flat nasal bridge, hypertelorism, low-set ears, and telecanthus. The phenotype of the patient was heterogeneous, hinge on the variable breakpoint and size [1, 2]. Some critical phenotypes could be explained by haploinsufficiency of major genes like *ANK3*, *HK1*, *PRKG1*. The *ANK3* gene encoded ankyrin-G, which was located mainly at the nodes of Ranvier and the axon initial segment (AIS), 2 subcompartments of neurons responsible for the generation of action potentials. The *HK1* gene encoded a ubiquitous form of hexokinase which localized to the outer membrane of mitochondria. Hexokinases phosphorylated glucose to produce glucose-6-phosphate, the first step in most glucose metabolism pathways. Mammals had three different isoforms of cyclic GMP-dependent protein kinase (Ialpha, Ibeta, and II). These isoforms acted as key mediators of the nitric oxide/cGMP signaling pathway and were important components of many signal transduction processes in diverse cell types. The *PRKG1* gene encoded the soluble Ialpha and Ibeta isoforms of PRKG by alternative transcript splicing. Genetic counseling for prenatal detection of 10q11-q22 interstitial deletions was difficult, owing to no ultrasound abnormalities could be detected in the affected fetus during pregnancy.

With the development of whole-exome sequencing (WES) and whole-genome mate-pair sequencing (WGMS) technology, increasing number of rare single nucleotide variations and copy number variations were being identified. However, to date, few cases of 10q11-q22 deletion syndrome with detailed phenotypes had been reported in the literature [3, 4]. In this study, we reported a patient with a de novo 20.76 Mb deletion on 10q11.23-q22.1. We described the phenotype of the patient in detail and reviewed reports of patients with similar 10q11-q22 interstitial deletions encompassing *ANK3*, *HK1* and *PRKG1*, and provided more information on the relationship between phenotypic characteristic and affected genes.

## Case presentation

The patient is the first child of healthy and non-consanguineous parents, born by vaginal delivery after full-term pregnancy with a birth weight of 2.8 kg. There were neither complications with pregnancy or delivery, nor history of jaundice, hypoxia and rescue after delivery. Apgar score at 5 min was 9.

**Fig. 1:**
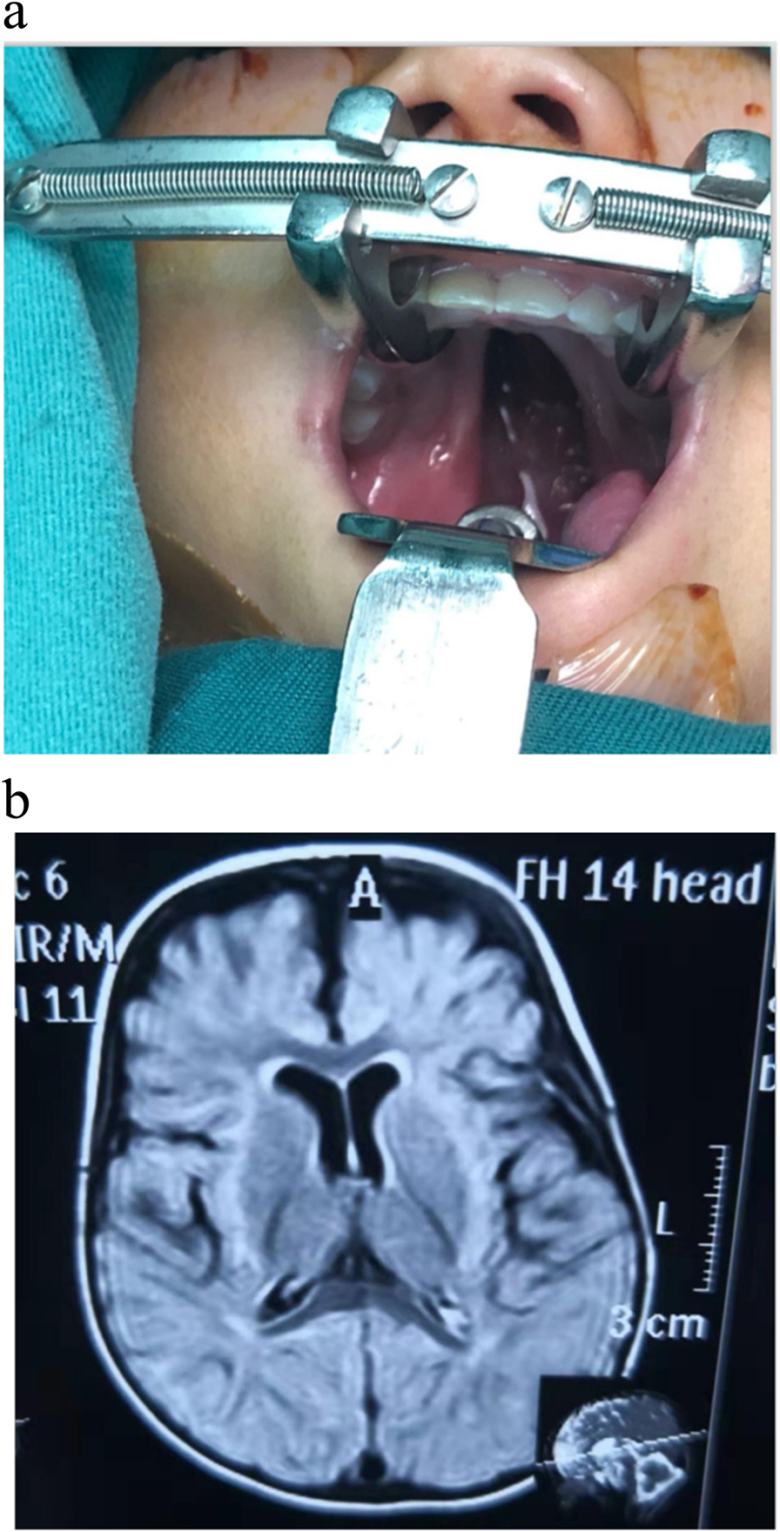
Phenotype of the patient. a The patient has a cleft palate. b MRI features of this patient

The patient was a 2 years old and 5 months girl when referred to our institution with a diagnosis of soft palate cleft, growth and development delay (Fig. 1a). Cleft palate was noticed after birth. The patient was not able to sit alone until she was 2 years old and was not able to climb until she was 2 years old and 5 months. At the age of 2 years and 5 months, she needed help to stand up and was unable to walk alone. Her language development was severely delayed and she could only speak simple words. Her weight was 7.5 kg (− 2 SD), length was 70 cm (− 2 SD), and occipitofrontal circumference (OFC) was 45 cm (− 2 SD, microcephalus). She showed distinctive features, including hypertelorism, epicanthal folds, flat nasal bridge, palpebral fissures, fibular bowing, foot asymmetry, brachydactyly. Her extremities were short, and her hands and feet were small. She had normal hearing evaluation and pursuit test result.

Cranial MRI revealed: 1. Corpus callosum was slightly thinner; 2. Bilateral frontotemporal external cerebral space was slightly wider, and local cerebral surface sulcus was slightly wider and deeper; 3. The anterior corners of lateral ventricles were slightly rounded (Fig. 1b).

We performed whole exome sequencing using the peripheral blood genomic DNA of the proband under the premise of informed consent. The WES library was captured using a biotinylated oligonucleotide probe library (Agilent SureSelect Human All exon v.6, Agilent). Sequencing was then performed on an Illumina HiSeq X-Ten platform (Illumina Inc., San Diego, CA, USA). Collect the sequencing raw data before the adapter sequence was removed and the low-quality reads were discarded. By Burrows-Wheeler Aligner, the filtered data was aligned to the human genome reference assembly (UCSC Genome Browser hg19). ANNOVAR software was used to annotate all variations [5]. Functional information was annotated using Exome Aggregation Consortium (ExAC), 1000 Genomes Project (1000G), Genome Aggregation Database (gnomAD), Human Gene Mutation Database (HGMD), ClinVar and OMIM. Revel, PolyPhen-2, SIFT and Mutation Taster were used to evaluate the effects of sequence variation on protein function. After the variants were analyzed and interpreted using the ACMG guidelines [6], none of these variants could account for the proband’s phenotype.

**Fig. 2:**
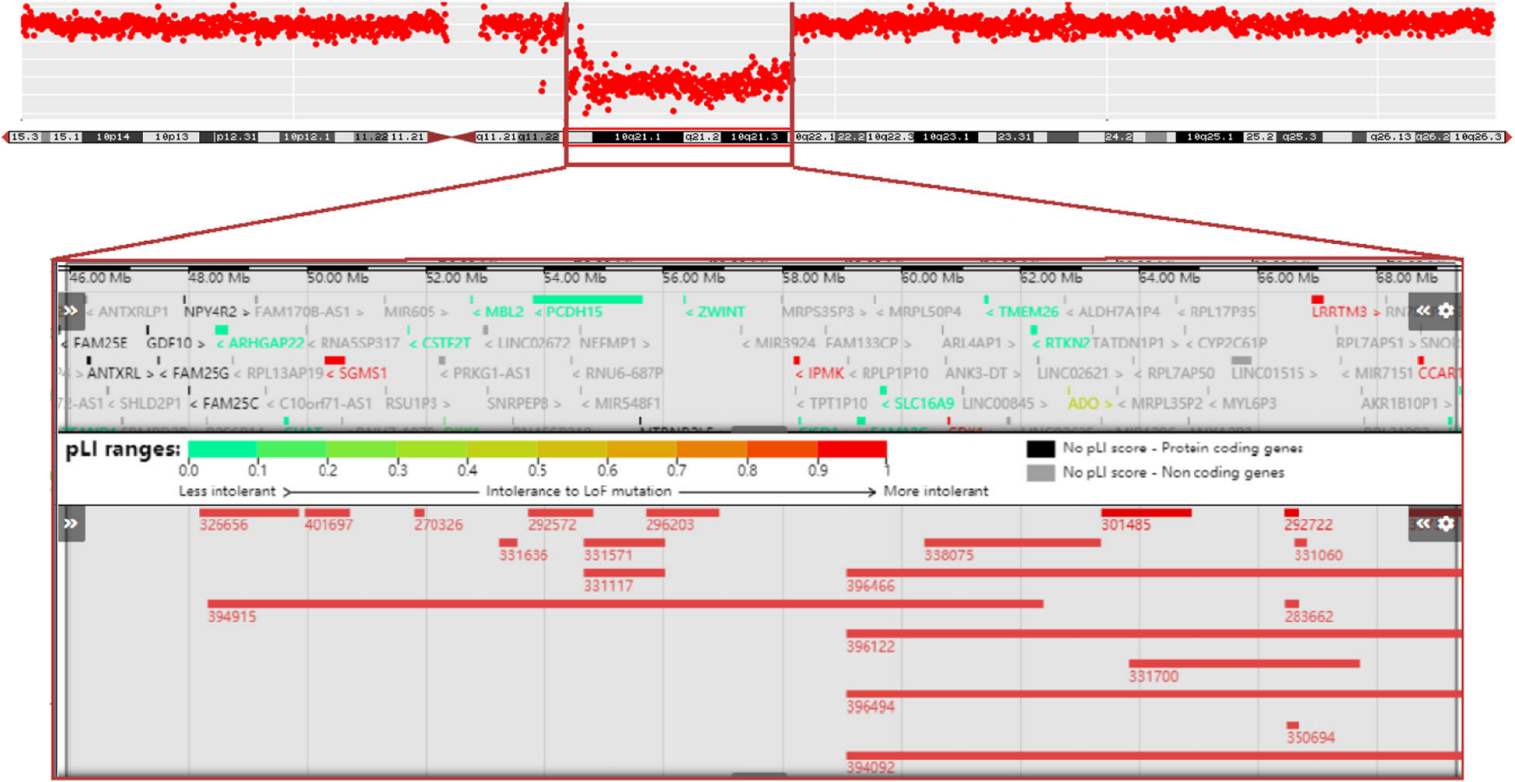
A de novo 20.76 Mb deletion on 10q11.23q22.1 was identified in the patient. Decipher data showed that the deletion encompasses 61 OMIM genes including *EGR2, ANK3, PRKG1, TFAM, BICC1, KIAA1279*, and showed the size and location of some patients with 10q11-q22 deletions. The deletion shown is detected by CNV-seq

**Fig. 3:**
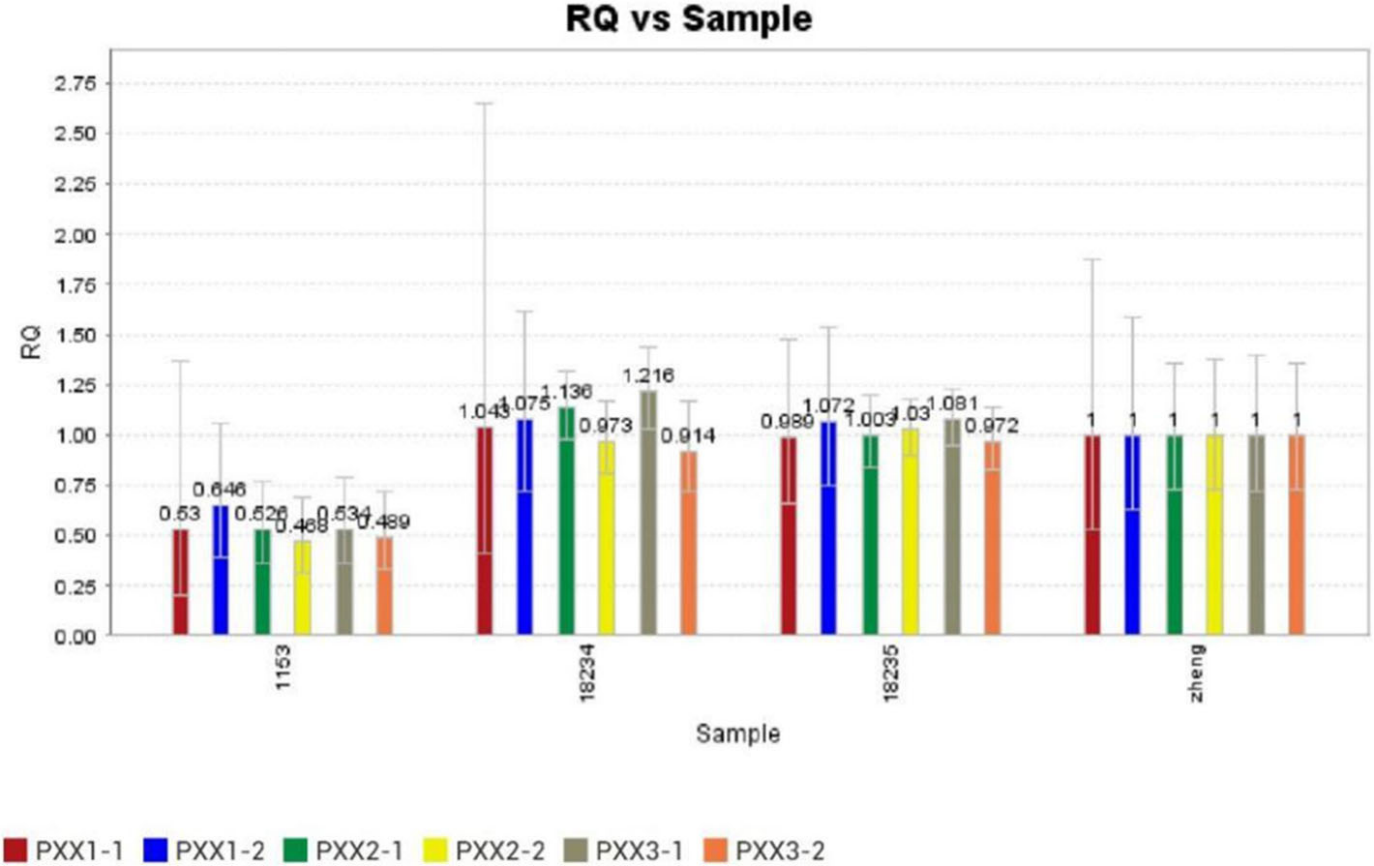
The CNV-Seq result was confirmed using qPCR. 1153: The patient; 18,234: The patient’s father; 18,235: The patient’s mother; zhang: controls

A non-size selected mate-pair library was constructed using the previously extracted DNA, and then BGISEQ-500 was used for 50-BP-END multiplex sequencing. Low-quality reads and reads containing sequencing adapters were removed, the high-quality pair-end reads were then aligned to the human genome reference assembly (UCSC Genome Browser hg19). We then reserved the uniquely mapped reads for subsequent analysis and the specific analysis method has been previously described in detail [7, 8]. Using this method, we were able to find all chromosome structural and numbers variations across the entire genome using uniquely paired reads, and find the corresponding break point of the chromosome. Chromosome breakpoints can be accurate to 1Kbp. Whole-genome mate-pair sequencing revealed a de novo 20.76 Mb deletion on 10q11.23-q22.1 (chr10:50,319,387-71,083,899). Decipher data showed that the deletion encompassed 61 OMIM genes including *EGR2, ANK3, PRKG1, TFAM, BICC1, KIAA1279*, and showed the size and location of some patients with 10q11-q22 deletions (Fig. 2). The deletion was not found in parents by qPCR (Fig. 3).

## Discussion and conclusion

Interstitial deletions of chromosome band 10q11-q22 was a genomic disorder characterized by developmental delay, congenital cleft palate, seizures, ventricular septal defect and muscular hypotonia, hypertelorism, broad-flat nasal bridge. It affected less than 1 in 50,000 infants. Patients had phenotypic heterogeneity, ranging from asymptomatic to severe birth defects, affected by breakpoint and deletion size. Sufficient cases could allow clinicians to better identify the disease. A few candidate genes were well identified like *ANK3*, *HK1* and *PRKG1*.

In our research, we described molecular and clinical findings in a female with soft palate cleft, growth and development delay. Using WES and WGMS, we revealed a 20.76 Mb interstitial deletion of the 10q11.23-q22.1 region containing 61 OMIM genes, which was probably responsible for the clinical phenotype of the patient. WES detected no pathogenetic variation associated with the patient’s clinical phenotype, which largely ruled out gene-level abnormalities. Notably, the pLI (probability of LoF intolerant) value of *PRKG1* and *ANK3* was 1, and the HI index (haploinsufficiency score) was 1% (0–10%). This suggested that deletions of these genes may have serious clinical symptoms. The PRKG1 proteins played a central role in regulating cardiovascular and neuronal functions in addition to relaxing smooth muscle tone, preventing platelet aggregation, and modulating cell growth [9]. The ANK3 proteins were believed to link the integral membrane proteins to the underlying spectrin-actin cytoskeleton and played key roles in activities such as cell motility, activation, proliferation, contact, and the maintenance of specialized membrane domains [10]. The pLI value of *HK1* was 0.83, and the loss of function of *HK1* mutation caused dominant inherited neurodevelopmental disorder with visual defects and brain anomalies (OMIM 618547) and could be the causes of patients’ neurological symptoms features.

A related clinical phenotype with deletion of 10q11-q22 had been described [11–15]. The first patient with 10q22 deletion was reported by Cook, who presented with developmental delay, distinctive features, and growth defects [1]. Ray et al. described the clinical phenotype of a 1-year-old boy with chromosome 10q11q21 deletion. The patient had limitation of joint movement, facial dysmorphism, significant developmental delay, mild hypotonia and intellectual disability [13]. MacDonald and Holden reported a case of a 9-year-old girl with chromosome 10q11.2q21 deletion. She had ptosis, cleft palate, mild hypotonia, developmental delay, mental retardation, seizures and deficit in language function [14]. Homoplastically et al. described a 5-year old girl with chromosome 10q11.2q22.1 deletion due to a t(10;13). The girl characterized by global developmental delay, strabismus, facial dysmorphism, ventricular septal defect, hypotonia, Hypoplasia of the optic nerve and fingers deformities [15]. Patients with deletion of 10q11-q22 were characterized by developmental delay, congenital cleft palate and muscular hypotonia. The phenotypes involved were heterogeneous, hinge on the variable breakpoints and size. Other variable phenotypes included limitation of joint movement, strabismus, including hypertelorism, epicanthal folds, flat nasal bridge, palpebral fissures, fibular bowing, foot asymmetry, brachydactyly, seizures, ventricular septal defect, and hypoplasia of the optic nerve. Unlike other reported cases, our patient did not have limitation of joint movement, seizures, ventricular septal defect, and hypoplasia of the optic nerve. Children with 10q11-q22 deletions greater than 20 MB had never been reported before, and we were the first to report and provide a detailed clinical phenotype, which brought further knowledge of 10q11-q22 deletions. In addition to the characteristics of developmental delay, congenital cleft palate and muscular hypotonia, our patients also showed distinctive features, including hypertelorism, epicanthal folds, flat nasal bridge, palpebral fissures, fibular bowing, foot asymmetry, brachydactyly, nanomelia.

Karyotyping in addition to chromosome microarray analysis (CMA) could also solve the problem. However, the WGMS had more accurate accuracy and resolution than karyotype analysis. It has been proposed that CMA was a stable and accurate platform for CNV detection. However, CMA technology could not analyze balanced translocations and complex rearrangements of chromosomes [16–18]. WGMS could solve all these problems at the same time. Recent research showed that the combination of WGMS and WES could analyze single nucleotide variation (SNV), small indel, CNV, balanced chromosomal translocation and complex rearrangement at one time and improved the diagnosis rate of rare diseases by low cover genome sequencing [19, 20]. Our results further demonstrate the clinical value of the combined Molecular diagnostic strategy of WGMS and WES in patients with genetic diseases and provided more information about the relationship between clinical features and interstitial deletions of chromosome band 10q11-q22.

## Data Availability

The datasets used and/or analyzed during the current study are available from the corresponding author on reasonable request.

## Abbreviations

WGMS: Whole-genome mate-pair sequencing
WES: Whole exome sequencing
qPCR: Quantitative real-time Polymerase chain reaction
CMA: Chromosomal microarray analysis
SNV: Single nucleotide variants
CNV: Copy number variations
OFC: Occipitofrontal circumference
pLI: Probability of LoF intolerant
MRI: Magnetic Resonance Imaging
